# Transdermal delivery of PeptiCRAd cancer vaccine using microneedle patches

**DOI:** 10.1016/j.bioactmat.2024.11.006

**Published:** 2024-11-19

**Authors:** Carmine D'Amico, Manlio Fusciello, Firas Hamdan, Federica D'Alessio, Paolo Bottega, Milda Saklauskaite, Salvatore Russo, Justin Cerioni, Khalil Elbadri, Marianna Kemell, Jouni Hirvonen, Vincenzo Cerullo, Hélder A. Santos

**Affiliations:** a Drug Research Program, Division of Pharmaceutical Chemistry and Technology, Faculty of Pharmacy, University of Helsinki, FI-00014, Helsinki, Finland; b Department of Pharmaceutical Biosciences, University of Helsinki, Faculty of Pharmacy ImmunoViroTherapy Lab, Drug Research Program, Viikinkaari 5, E00790, Helsinki, Finland; c Department of Chemistry, Faculty of Science, University of Helsinki, FI-00014, Helsinki, Finland; d Helsinki Institute of Life Science (HiLIFE), University of Helsinki, Fabianinkatu 33, 00710, Helsinki, Finland; e Translational Immunology Program (TRIMM), Faculty of Medicine Helsinki University, University of Helsinki, Haartmaninkatu 8, 00290, Helsinki, Finland; f Digital Precision Cancer Medicine Flagship (iCAN), University of Helsinki, 00014, Helsin-ki, Finland; g Department of Molecular Medicine and Medical Biotechnology and CEINGE, Naples University Federico II, 80131, Naples, Italy; h Department of Biomaterials and Biomedical Technology, The Personalized Medicine Research Institute (PRECISION), University Medical Center Groningen (UMCG), University of Groningen, 9713 AV, Groningen, the Netherlands

**Keywords:** Microneedles, Cancer therapy, Adenoviral vector, Vaccine

## Abstract

Microneedles (MNs) are a prospective system in cancer immunotherapy to overcome barriers regarding proper antigen delivery and presentation. This study aims at identifying the potential of MNs for the delivery of Peptide-coated Conditionally Replicating Adenoviruses (PeptiCRAd), whereby peptides enhance the immunogenic properties of adenoviruses presenting tumor associated antigens. The combination of PeptiCRAd with MNs containing polyvinylpyrrolidone and sucrose was tested for the preservation of structure, induction of immune response, and tumor eradication. The findings indicated that MN-delivered PeptiCRAd was effective in peptide presentation *in vivo,* leading to complete tumor rejection when mice were pre-vaccinated. A rise in the cDC1 population in the lymph nodes of the MN treated mice led to an increase in the effector memory T cells in the body. Thus, the results of this study demonstrate that the combination of MN technology with PeptiCRAd may provide a safer, more tolerable, and efficient approach to cancer immunotherapy, potentially translatable to other therapeutic applications.

## Highlights

• PVP-Sucrose MNs preserve and release infective viral particles effectively.
• MN delivery of PeptiCRAd shows complete tumor rejection in murine models.
• Enhanced cDC1 population correlates with effective antigen presentation in MNs.
• MN-based PeptiCRAd vaccines elicit strong effector memory T-cell responses.

## Graphical abstract

**Figure:**
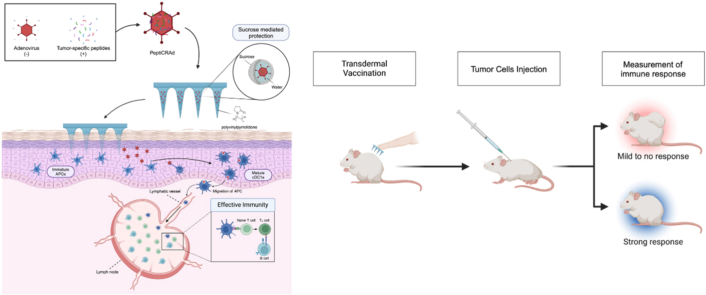

## 1 Introduction

Immunotherapy is the most advanced and rapidly growing treatment modality in oncology that focuses on the modulation of host immune response for the recognition and destruction of cancer cells [1]. Some of the recent clinical trials have shown that cancer vaccines are useful but there is a difficulty in ensuring that the right antigen reaches the right cell [[2], [3], [4], [5]].

Microneedles (MNs) are one of the non-invasive methods that can easily pierce the skin barrier and have attracted much interest for their compliance [6,7]. Not only do these devices present antigens to the skin's immunologically active compartments but they have also been demonstrated to encapsulate and deliver both traditional and novel vaccine components to promote immunity [[8], [9], [10], [11], [12]]. For instance, hyaluronate-based MNs have been shown to effectively deliver conjugated antigenic peptides for prophylactic cancer immunotherapy, enhancing antigen-specific immune responses in melanoma models [13]. Their minimal pain and ease of self-administration enhance patient compliance, which is crucial for consistent and effective vaccination, particularly in populations with needle phobia or in resource-limited settings [[14], [15], [16]]. This high compliance also reduces the burden on healthcare systems by enabling safe and efficient self-administration of vaccines and treatments [17,18].

On these grounds, here we have created a MN patch encapsulating Peptide-coated Conditionally Replicating Adenoviruses (PeptiCRAd) which can be considered as an advanced approach to oncolytic virus-based cancer vaccines. Specifically, PeptiCRAd enhances the adjuvant effect of adenoviruses and combines them with tumor associated antigens (TAAs) to control the immune response reprogramming and orchestrating cancer specific T cells [19,20]. This approach does not only enhance the direct delivery and presentation of the mechanisms but also focus the immune system to the tumor associated antigens rather than the viral components. The synergy between the enhanced antigen presentation and targeted immune response increases the efficacy of CD8^+^ T cell mediated anti-tumour attacks, which is crucial as standard therapies often fail to distinguish between antiviral and antitumour responses. In addition, the used PeptiCRAd technology is already in the phase I clinical trial (registered at Clinicaltrials.gov: NCT 05492682) [21].

The incorporation of PeptiCRAd with MN technology requires encapsulation with a specific ratio of polyvinylpyrrolidone (PVP) and sucrose to enhance the delivery of the vaccine. PVP is crucial in the structural stability and functionality [[22], [23], [24], [25], [26]] of MNs and sucrose is important in maintaining the infectivity of the virus after the production process [[27], [28], [29], [30]]. Initial *in vitro* tests were carried out to ensure that the adenovirus was stable and operational after its encapsulation and delivery. Additional *in vivo* experiments showed the level of immune response elicited by both MN and subcutaneous delivery of PeptiCRAd, as well as the complete tumor rejection for B16.OVA engraftment in mice vaccinated with PeptiCRAd loaded in MN. Immunophenotyping studies showed a significant rise in Conventional Type 1 Dendritic Cells (cDC1), which are involved in the activation of T cells for tumor killing together with a rise in Effector Memory cells and IFN-g expressing CD8 T-cells [31,32]. These findings not only confirm the possibility of using PeptiCRAd technology in combination with MNs but also demonstrate solutions to the main challenges associated with the delivery of vaccines, presentation of antigens, and patients’ compliance with the prescribed therapy. This study also confirms that MNs and PeptiCRAd are feasible and can be combined in transdermal cancer immunotherapy, which supports their further research and trials.

## 2 Materials and methods

### 2.1 Fabrication of MNs

A polymer solution was prepared using 10 % PVP and 10 % sucrose (20 % total concentration). The PeptiCRAd suspension was first centrifuged at 4000 rpm and 5 °C for 1 h. The resulting PeptiCRAd suspension was then dispensed into MN molds (Micropoint Technologies, Singapore) featuring dimensions of 800 μm height, 200 μm base, and 500 μm pitch and centrifuged again at 4000 rpm and 5 °C for 1 h to facilitate needle formation. Following this, the molds were left to dry at ambient room temperature for 24 h to ensure complete solvent removal and solidification of the MN structure. To form a stable base and pedestal for the MNs, a PVP aqueous solution (20 % w/v) was added, and a final round of centrifugation under the same conditions secured the PVP base to the needles. An additional overnight drying period at room temperature ensured the complete setting of the microneedles. The samples were then stored in a desiccator.

### 2.2 Scanning electron microscopy imaging

For detailed examination of the surface morphology of the MNs, a Hitachi S-4800 Scanning Electron Microscope (SEM) was employed. To prepare the MN samples for SEM analysis, they were first coated with a 5 nm layer of Au/Pd alloy using a Cressington 208HR sputter coater to enhance conductivity and image clarity.

### 2.3 Mechanical properties of MNs

The mechanical characteristics of the MNs were assessed using a CT3 Texture Analyzer (Brookfield, Canada) set in compression mode. The initial heights of the MNs were measured using an EVOS XL optical microscope (Invitrogen, USA). The MNs were then adhered to the texture analyzer's probe using double-sided tape and compressed against a flat aluminum platform at a speed of 0.5 mm s^−1^. The mechanical resistance of the MNs during axial compression was analyzed, with force per needle plotted against deformation.

### 2.4 Piercing ability assessment

The piercing abilities of the MNs were tested using Parafilm M (Amcor, USA), which was folded to create an eight-layer film approximately 1 mm in thickness. The MNs were attached to the texture analyzer's probe and lowered onto the Parafilm M at 0.5 mm s^−1^, exerting a force of 30 N for 30 s. The number of perforations in each layer was counted using the optical microscope. Additionally, the MNs were inserted into *ex vivo* pig skin for 30 s using the Texture Analyzer, and SEM images were taken before and after insertion to assess any morphological changes.

### 2.5 Oncolytic adenoviruses for *in vitro* and *in vivo* experiments

To assess fitness and expression of the transgene after microneedle encapsulation, we used the oncolytic Ad5-D24-RFP virus. This adenovirus has been previously described in the literature [33,34]. For animal studies, the oncolytic murine virus VALO-mD90143 was employed. VALO-mD901 is a conditionally replicating adenovirus of the chimeric 5/3 serotype with a 24-base pair deletion in the E1A region (Ad5/3-D24). Additionally, the adenoviral E3 region was replaced by an expression cassette consisting of the human CMV promoter, followed by murine OX40L and murine CD40L genes separated by a 2A self-cleaving peptide sequence, and ending with a β-rabbit globin polyadenylation signal. The generation of VALO-mD901 followed methodologies previously outlined in the literature [35].

### 2.6 Peptides

The short and poly-K murine peptides used in this study were purchased from Chempeptide Limited (Shanghai, China). The specific peptides utilized were SIINFEKL (OVA) and KKKKKKSIINFEKL (OVA). The modified version, KKKKKKSIINFEKL, includes an additional six lysine (K) residues at the N-terminus, which increases its positive charge. This poly-lysine modification enhances binding to negatively charged surfaces.

### 2.7 PeptiCRAd complex

PeptiCRAd complexes were prepared as previously described [21]. Briefly, the complexes were formed by mixing the VALO-mD901 virus with peptides containing a poly-K tail in PBS, at a ratio of 20 μg of peptides per 1 × 10^9^ VP per mouse. The mixture was incubated at room temperature for 15 min to ensure proper binding.

### 2.8 Viability assay

MTS assay was performed on A549 cell line according to the manufacturer's protocol (CellTiter 96 AQueous One Solution Cell Proliferation Assay; Promega, Nacka, Sweden). Spectrophotometric data were acquired with Varioskan LUX (Thermo Scientific, Carlsbad, CA, USA).

### 2.9 Cross-presentation experiment

Murine dendritic JAWS-II cells were infected with dissolved MN carrying PeptiCRA-SIINFEKL, with one well left uninfected as a control. After overnight incubation, the cells were gently detached using 10 mM EDTA and stained. Staining was preceded by blocking Fc receptors with anti-mouse CD16/32 (101320; BioLegend) for 15 min. This was followed by staining with a mixture of antibodies: APC-H2Kb-bound SIINFEKL (141606; BioLegend) and PerCP/Cyanine5.5 anti-mouse CD86 (105028; BioLegend). Stained samples were acquired using a BD Accuri 6C Plus Flow Cytometer (BD Biosciences) and flow cytometric data were analyzed using FlowJo software v.10 (BD Life Sciences).

### 2.10 Cell lines

The murine dendritic cell line JAWSII was cultured in alpha MEM supplemented with 20 % FBS, 1 % glutamine, 100 μg/ml streptomycin, and 100 U/ml penicillin. B16.OVA, a mouse melanoma cell line expressing chicken OVA, was kindly provided by Professor Richard Vile (Mayo Clinic, Rochester, MN, USA). B16.OVA cells were cultured in RPMI with 10 % FBS, 1 % L-glutamine, 1 % penicillin/streptomycin, and 5 mg/mL Geneticin (Life Technologies). The cells were maintained at 37 °C with 5 % CO2 in a humidified atmosphere.

### 2.11 Murine IFN-γ ELISpot assay

IFN-γ ELISpot assays were conducted using commercially available mouse ELISpot reagent sets (ImmunoSpot, Bonn, Germany), following the manufacturer's instructions. At the experiment's endpoint, spleens were collected and processed into a single-cell suspension by passing them through a 70 μm strainer using the back of a syringe plunger, with anti-aggregate reagent (CTL Anti-Aggregate Wash, Cat# CTL-AA-005) from the same manufacturer. Red blood cells were lysed using ACK buffer (Gibco) according to the manufacturer's instructions. The resulting splenocytes were resuspended in CTL test medium (ImmunoSpot, Bonn, Germany) and counted. For each well, 5 × 10⁵ splenocytes were seeded and stimulated with 20 ng/μl (2 μg per well in total) of each peptide at 37 °C for 72 h. After 3 days of stimulation, the plates were developed, and spot counts were obtained using the CTL ImmunoSpot ELISpot plate reader system (ImmunoSpot, Bonn, Germany).

### 2.12 Animal experiments

Female C57BL/6 mice (4 to 6-week-old) were purchased from Envigo (Laboratory, Bar Harbor, Maine, UK). Mice were housed in individually ventilated cages (IVC) with a maximum of 5 mice per cage, provided with food and water ad libitum, and maintained on a 12-h light/dark cycle. Mice were monitored daily by animal caretakers. Patch application and tumor measurements were performed under isoflurane anesthesia (AttaneTM Vet). Euthanasia was conducted using CO2, followed by neck dislocation to confirm death.

For the pre-immunization experiments, mice were immunized three times on days 0, 1, and 2. Twenty-one days after the last vaccination, mice were boosted with another patch application. One week after boosting, mice were sacrificed, and spleens and lymph nodes were collected.

In the tumor treatment experiment, the same immunization schedule was followed, with tumor implantation of murine B16.OVA one week after the priming phase. Mice were boosted with an intratumoral injection three weeks after the last patch administration of the priming phase. Tumor growth was recorded using a caliper, and mice were sacrificed on day 27. Spleens and lymph nodes were collected and reduced to single-cell suspensions for immunophenotyping and ELISpot assay.

For tumor engraftment, 3 × 10^5^ B16.OVA cells were injected into the right flank of the mice, which had been previously shaved. A total of 1 × 10^9^ VP of VALO-mD901 virus was injected intratumorally in a final volume of 50 μl per mouse for the boosting dose. Tumor growth was monitored using a digital caliper to measure the vertical and horizontal dimensions of each tumor every two days. Tumor volume was calculated using Equation (1):

(1) $$\text{Tumor}\;\text{Volume}=\frac{\text{long}\;\text{measure}\;x\;{\left(\text{short}\;\text{measure}\right)}^{2}}{2}$$

All animal experiments were reviewed and approved by the Experimental Animal Committee of the University of Helsinki and the Provincial Government of Southern Finland (license numbers ESAVI/11895/2019 and ESAVI/12722/2022). The maximal tumor size/burden allowed by our ethical permit is 18 mm in diameter. This limit was never exceeded in any of the experiments conducted in this study.

### 2.13 Flow cytometry analysis

Upon processing single-cell suspensions from spleens and lymph nodes, cells were centrifuged at 300×*g* for 5 min and washed twice with PBS. Each sample was then incubated with 1 μg of TruStain FcX™ (anti-mouse CD16/32) Antibody (BioLegend) for 15 min according to the manufacturer's instructions. Subsequently, cells were stained with fluorochrome-labeled antibodies and incubated on ice for 30 min, protected from light. Stained cells were washed twice with PBS before acquisition.

The antibodies used in this study included BV711 CD3 (clone: 145-2C11, cat: 563123, BD Biosciences, 0.5μl/1 million cells), BV510 CD8 (clone: 53-6.7, cat: 563068, BD Biosciences, 1μl/1 million cells), PE-CF594 CD4 (clone: RM4-5, cat: 562285, BD Biosciences, 0.5μl/1 million cells), APC CD62L (clone: MEL-14, cat: 17-0621-81, eBioscience, 0.5μl/1 million cells), AF700 CD44 (clone: IM7, cat: 103026, BioLegend, 1μl/1 million cells), v450 CCR7 (clone: 4B12, cat: 560805, BD Biosciences, 1μl/1 million cells), BV650 TNF-α (clone: MP6-XT22, cat: 506333, BioLegend, 1μl/1 million cells), PE-Cy7 IL-2 (clone: JES6-5H4, cat: 503832, BioLegend, 1μl/1 million cells), PE IFN-γ (clone: XMG1.2, cat: 505808, BioLegend, 1μl/1 million cells), BV711 CD11b (clone: M1/70, cat: 101241, BioLegend, 0.5μl/1 million cells), FITC CD11c (clone: N418, cat: 25-0114-82, eBioscience, 0.5μl/1 million cells), PerCP/cy5.5H2-Kd (clone: AF6-88S, cat: 116516, BioLegend, 1μl/1 million cells), PE CD103 (clone: 2E7, cat: 121406, BioLegend, 1μl/1 million cells), APC PDCA (clone: 927, cat: 127015, BioLegend, 1μl/1 million cells), APC anti-mouse CD3 (clone: 17A2, cat: 100236, BioLegend, 0.5 μg/1 million cells), FITC anti-mouse CD8a (clone: 53-6.7, cat: 100706, BioLegend, 1 μg/1 million cells), PerCP/cy5.5 anti-mouse CD107a (LAMP-1) (clone: 1D4B, cat: 121625, BioLegend, 5 μl/1 million cells), PE anti-mouse CD279 (PD-1) (clone: 29 F.1A12, cat: 135206, BioLegend, 1 μg/1 million cells), PerCP/cy5.5 anti-mouse CD366 (Tim-3) (clone: RMT3-23, cat: 119718, BioLegend, 0.5 μg/1 million cells), PE-conjugated antihuman HLA-A, HLA-B, and HLA-C (clone: W6/32, cat: 311406, BioLegend, 5 μl/1 million cells), and APC anti-mouse H2-Kd (clone: SF1-1.1, cat: 116619, BioLegend, 0.25 μg/1 million cells). All antibody mixes were incubated in a final volume of 100 μl and generally used according to their respective manufacturer instructions.

Stained samples were acquired using a BD LSRFortessa™ Cell Analyzer (BD Biosciences), and flow cytometric data were analyzed using FlowJo software v.10 (BD Life Sciences).

### 2.14 Statistical analysis

Statistical analysis was performed using GraphPad Prism 10.0 software (GraphPad Software, USA) or Python. All results are expressed as the mean ± SEM. Additional information on the statistical tests used can be found in the corresponding figure legend.

## 3 Results

### 3.1 Mechanical integrity and piercing ability of PVP-sucrose MNs for transdermal delivery

**Fig. 1:**
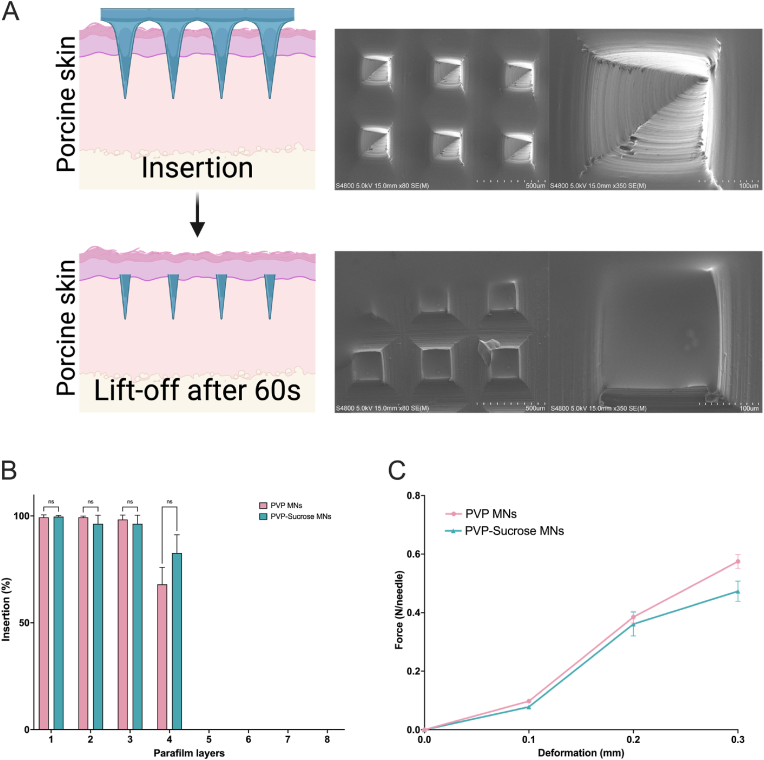
**Characterization and Mechanical Assessment of PVP-Sucrose MNs.** (A) Assessment of MN detachment from the base after 60-s insertion into *ex vivo* porcine skin, demonstrating the dissolution of the MN tips by SEM. (B) Comparison of piercing abilities between PVP and PVP-Sucrose MNs through multiple layers of Parafilm (approximately 100 μm thick each) to ensure that sucrose addition does not compromise mechanical properties or penetration capability. (C) Force-deformation curves for both PVP and PVP-Sucrose MNs, indicating similar mechanical integrity and elasticity, confirming that sucrose addition does not affect the MNs' mechanical suitability for transdermal applications.

After preparing the MNs using controlled temperature micro-molding (Fig. S1), we characterized their morphology with scanning electron microscopy (SEM). We also assessed the detachment of the MNs from the base after a 60-s insertion into *ex vivo* porcine skin (Fig. 1A). This experiment aimed to develop a formulation capable of delivering the PeptiCRAd vaccine quickly for subsequent *in vivo* experiments.

To ensure that adding sucrose to the PVP mixture does not compromise the mechanical and piercing properties of the MNs we conducted a series of additional tests. We compared the piercing abilities of both PVP and PVP-Sucrose MNs through multiple layers of Parafilm [36], each approximately 100 μm thick (Fig. 1B). The results indicate that both MN formulations consistently achieve high levels of insertion through the first three layers of Parafilm, maintaining significant insertion at the fourth layer. We also performed the same experiment after loading PeptiCRAd within the MNs, observing no impact on the properties (Fig. S2). This MN-performance suggests that sucrose does not compromise the structural integrity or piercing ability of the MNs. The ability to effectively penetrate up to four layers of Parafilm, cumulatively around 400 μm, indicates their potential to breach the stratum corneum, the primary barrier to topical drug delivery. The stratum corneum, measuring 10–15 μm thick, is composed of dead flattened corneocytes surrounded by a lipid extracellular matrix. Below it lies the viable epidermis, a cellular, avascular tissue measuring 50–100 μm thick. Together, these layers form the epidermis [37]. The demonstrated penetration through several hundred micrometers of Parafilm underscores the MNs' capability for effective transdermal delivery, reaching the dermis where a rich capillary bed in the superficial dermis just below the epidermis facilitates drug uptake into systemic circulation.

Both MN formulations show a similar initial phase where minimal force is required for low levels of deformation, suggesting comparable structural integrity at the outset (Fig. 1C). As deformation increases, both types of MNs demonstrate a progressive rise in the force required for further deformation, indicative of inherent material elasticity and robustness essential for effective skin penetration without breaking [38]. Importantly, the force-deformation curves of the PVP-Sucrose MNs closely mirror those of the pure PVP MNs, indicating that the addition of sucrose does not detrimentally affect the mechanical properties of the MNs. This finding supports the use of sucrose in MN formulations, suggesting it enhances stability or delivery efficiency without impacting their mechanical suitability for transdermal applications.

### 3.2 Preservation of viral infectivity in MN formulations with sucrose

**Fig. 2:**
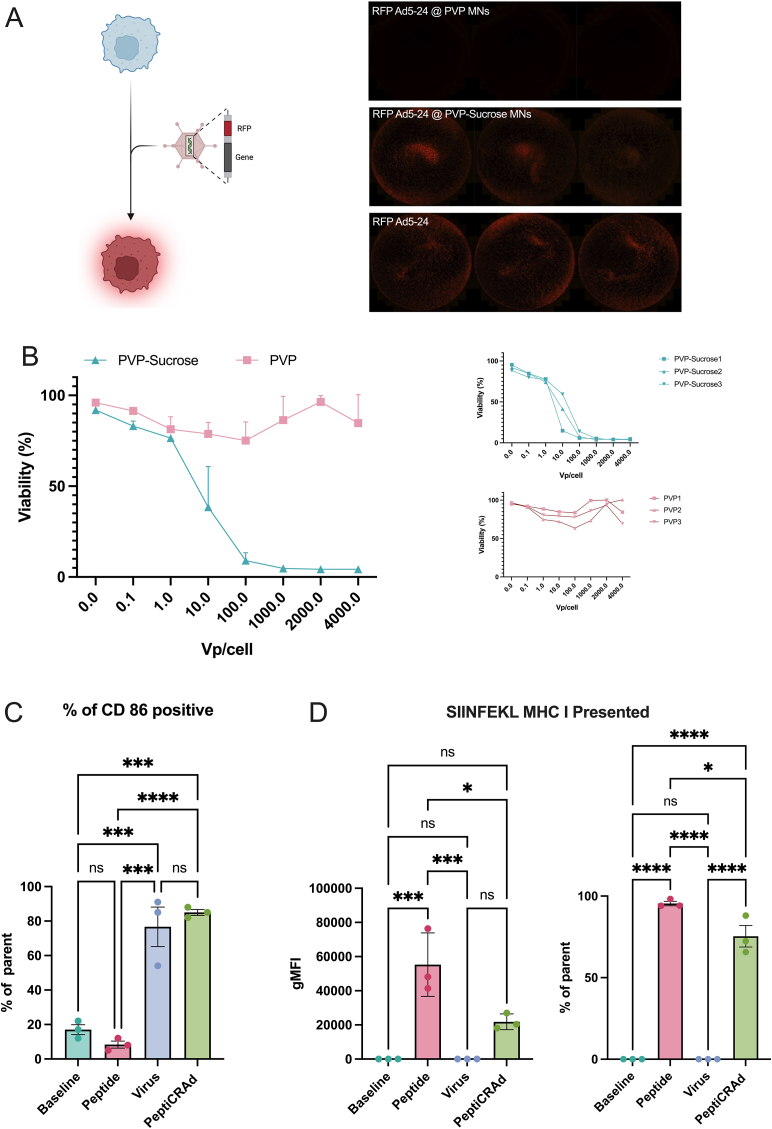
**Assessment of Immune Activation by PVP-Sucrose MNs.** (A) Schematic representation of the adenovirus infection leading to RFP expression. RFP expression in A549 cells treated with solutions from PVP MNs, PVP-Sucrose MNs, and direct virus application. (B) Cell viability assay demonstrating the effects of viral particle concentration on cell viability. (C) Flow cytometry analysis of CD86 expression in JAWS II dendritic cells, showing significantly higher levels in cells treated with Virus MNs and PeptiCRAd MNs. (D) Presentation of SIINFEKL peptide on MHC class I molecules, observed in Peptide MNs and PeptiCRAd MNs but absent in Baseline and Virus MN groups. ANOVA was used for statistical analysis, and p-values were set at probabilities ∗p < 0.05, ∗∗p < 0.01, ∗∗∗p < 0.001, and ∗∗∗∗p < 0.0001.

Upon infecting A549 lung carcinoma cells, the engineered Ad5–24 virus prompts the cells to produce red fluorescent protein (RFP) (Fig. 2A). This experiment aimed to assess the virus’ viability when encapsulated within PVP and PVP-Sucrose MN-formulations. After dissolving the MNs, cells treated with the solution from PVP MNs showed no fluorescence. This indicates a loss of viral infectivity, likely due to the drying process used in making the PVP MNs. Conversely, cells treated with the solution from PVP-Sucrose MNs exhibited clear fluorescence. This suggests that sucrose effectively protects viral particles during processing, preserving their infectivity.

To validate the fluorescence findings, a CellTiter-96® AQueous Non-Radioactive Cell Proliferation Assay (MTS) assessed cell viability across various concentrations of viral particles from both MN formulations (Fig. 2B). After five days, cells exposed to viral particles from PVP MNs showed nearly 100 % viability. This indicates no effective viral infection. In stark contrast, cells exposed to increasing concentrations of viral particles from PVP-Sucrose MNs exhibited a significant drop in viability. Viability fell below 50 % at 10 vp/cell, with extensive cell death at higher concentrations. This confirms that PVP-Sucrose MNs not only preserve but also effectively release infective viral particles, substantially impacting cell viability.

### 3.3 Immune response activation by PeptiCRAd-loaded MNs

To understand how the components of PeptiCRAd affect immune activation, we tested four different types of PVP-Sucrose MNs. We had Baseline MNs (our control group with no active agents), Peptide MNs (loaded only with the SIINFEKL peptide), Virus MNs (containing only the adenovirus), and PeptiCRAd MNs (which combined the SIINFEKL peptide and the adenovirus). We used JAWS II dendritic cells, which are a model for immature murine dendritic cells (DCs), to assess how well these MNs could activate the immune system and mimic presentation of the peptides displayed by MHC I molecules.

We performed flow cytometry to analyze CD86 expression, an important marker for dendritic cell activation. CD86 levels were significantly higher in cells treated with Virus MNs and PeptiCRAd MNs, indicating effective stimulation by the adenovirus (Fig. 2C). Next, we looked at the presentation of the SIINFEKL peptide on MHC class I molecules, which is crucial for T-cell recognition and response (Fig. 2D). The baseline and Virus MN groups showed no peptide presentation, while the Peptide MNs and PeptiCRAd MNs showed strong peptide presentation. Interestingly, the Peptide MNs had higher levels of MHC class I-bound peptide compared to PeptiCRAd MNs. This difference is likely because there were no competing adenoviral components in the Peptide MNs. These findings show that each component plays a specific role in modulating the immune response.

The balanced viral adjuvant effect and SIINFEKL peptide presentation achieved with PeptiCRAd encapsulated in PVP-Sucrose MNs supports the efficacy of this delivery platform, paving the way for further *in vivo* studies to explore its therapeutic potential.

### 3.4 Immunogenicity of PeptiCRAd via transdermal and subcutaneous delivery

**Fig. 3:**
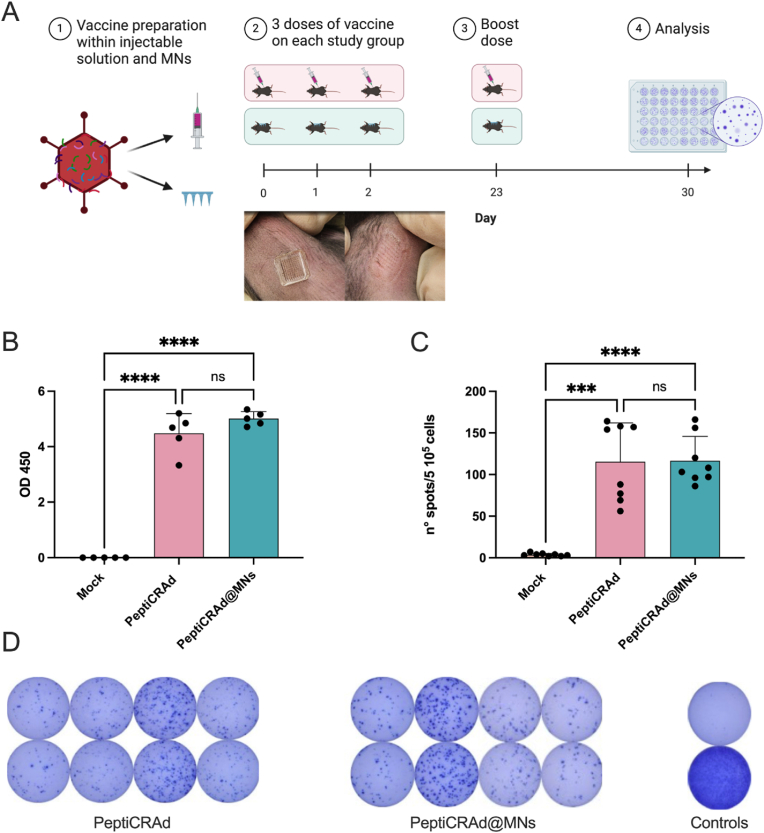
**Immunogenicity of PeptiCRAd Delivered via MNs and Subcutaneous Injection.** (A) Schematic representation of the *in vivo* experiment. (B) ELISA results measuring Ig specific for the adenovirus component of PeptiCRAd. (C) ELISpot assay results focusing on the T-cell response. (D) Visualization of the ELISpot assay showing the frequency of T-cells activated in response to the SIINFEKL peptide. The MN group displayed a higher density of spot-forming cells, indicative of robust T-cell activation. Statistical analysis was conducted using ANOVA, with p-values set at ∗p < 0.05, ∗∗p < 0.01, ∗∗∗p < 0.001, and ∗∗∗∗p < 0.0001.

To evaluate the immunogenicity of PeptiCRAd delivered via MNs compared to subcutaneous injection, we conducted an *in vivo* study with three groups: one group receiving PeptiCRAd via MNs, another group via subcutaneous injection, and a third control group that received no treatment. Prior to administration, we quantified potential losses using an immunocytochemistry (ICC) assay and adjusted the loading by adding an excess of PeptiCRAd to ensure that the MN group received a comparable dose to the subcutaneous injection group. Both treated groups received identical dosages and followed the same administration schedule. (Fig. 3A). The ELISA results measuring immunoglobulin (Ig) specific for the adenovirus component of PeptiCRAd (Fig. 3B) showed that both the MN and subcutaneous delivery groups exhibited significantly elevated Ig levels compared to the control, demonstrating successful induction of an antibody-mediated immune response against the adenovirus in both delivery methods. The skin's layers, rich in antigen-presenting cells (APCs) such as Langerhans and dermal dendritic cells, provide direct access to these immune cells upon MN penetration. This interaction enhances antigen capture, processing, and presentation, leading to an intensified humoral immune response.

Further immunological assessment focused on the T-cell response, specifically on the presentation of the SIINFEKL peptide, an indicator of cellular immunity. The response was analyzed using ELISpot (Fig. 3C), which measures T-cell activation by detecting spot-forming cells responsive to the SIINFEKL peptide. Comparative results demonstrated that both delivery methods effectively stimulated a T-cell response, with MNs showing a higher frequency of spot-forming cells. This suggests enhanced antigen processing and presentation through the dermal delivery route facilitated by MNs. Images from the ELISpot assay in Fig. 3D visually represent the frequency of T-cells activated in response to the SIINFEKL peptide, displaying a higher density of spot-forming cells in the MN group, indicative of robust T-cell activation.

The combined findings from these assays demonstrate that PeptiCRAd via MNs can effectively initiate both humoral and cellular immune responses. Specifically, the T-cell activation is not perturbated by MNs delivery, demonstrating the capability of MNs not only to deliver the vaccine effectively but also to induce a robust immunological response. Thus, supports their use as a viable alternative to traditional injection methods in cancer immunotherapy strategies.

### 3.5 Tumor rejection and immune activation by PeptiCRAd vaccination using MNs

**Fig. 4:**
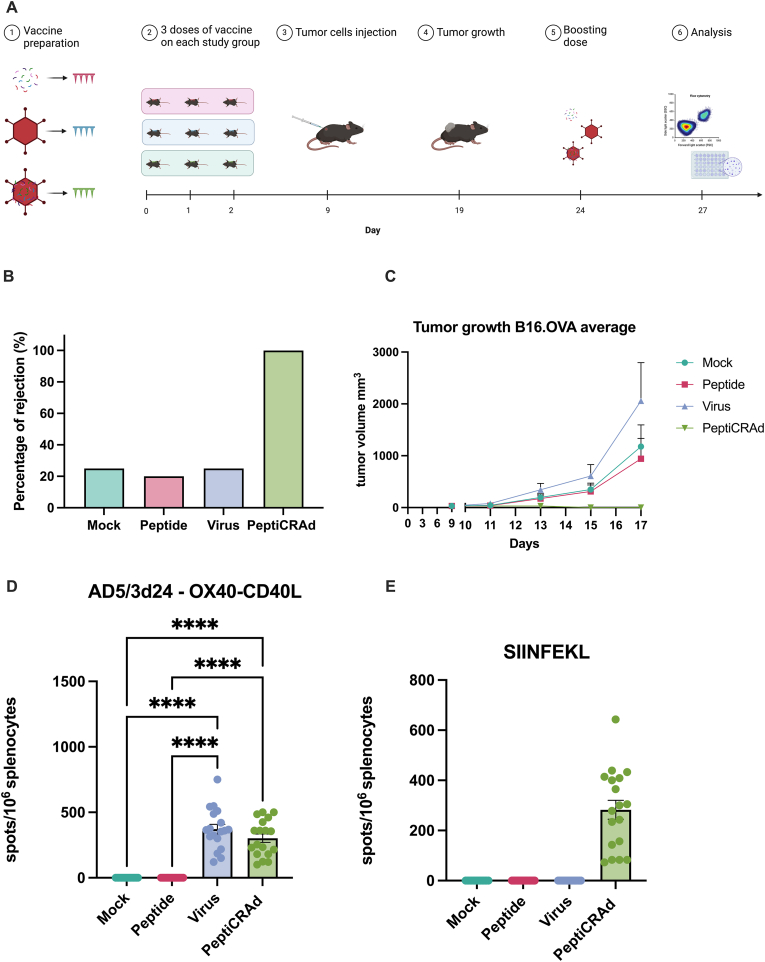
**Evaluation of PeptiCRAd Delivered via MNs in Murine Melanoma Model.** (A) Schematic representation of the *in vivo* experiment setup, including vaccine preparation, dosing schedule, tumor cell injection, tumor growth monitoring, boosting dose administration, and analysis. (B) Tumor rejection data showing complete tumor rejection in the PeptiCRAd group. (C) Average tumor growth curves for each treatment group. (D) ELISpot assay results measuring T-cell responses against the AD5/3d24 - OX40-CD40L antigen. (E) ELISpot assay results measuring IFN-g as T-cell responses against the SIINFEKL peptide. Statistical analysis was conducted using ANOVA, with p-values set at ∗p < 0.05, ∗∗p < 0.01, ∗∗∗p < 0.001, and ∗∗∗∗p < 0.0001.

To assess the potential immune effect of the vaccine system developed here, we pre-vaccinated the mice bearing B16.OVA murine melanoma. We decorated PeptiCRAd with the SIINFEKL peptide, which is the immunogenic part of the chicken albumin protein [39]. We divided the mice in four groups: one mock group, and three groups receiving MN formulations with either Peptide (SIINFEKL), Virus (adenovirus), or PeptiCRAd. Mice were given three doses (20 μg of peptides per 1 × 10^9^ viral particles) of their respective treatments via MNs, followed by an injection of B16.OVA tumor cells. Tumor growth was monitored, and a booster dose for T-cell recall was administered after three weeks from the last priming dose, mirroring clinical approach (Fig. 4A). Engraftment data (Fig. 4B) showed that the PeptiCRAd group exhibited complete tumor rejection, with all mice maintaining minimal to no detectable tumor growth throughout the study. This finding underscores the superior efficacy of the PeptiCRAd vaccine delivered via MNs in preventing tumor development, highlighting its potential as a successful cancer vaccination strategy. Looking at average tumor growth across different treatment groups (Fig. 4C), the PeptiCRAd group showed negligible tumor growth compared to the Mock, Peptide, and Virus groups. Tumor volumes in the Mock, Peptide, and Virus groups increased steadily over the 17-day observation period while the PeptiCRAd group maintained tumor volumes close to 0 mm³, indicating effective tumor suppression. Upon animal sacrifice, ELISpot assay on resected spleenocytes provided a quantitative measure of T-cell responses to the treatments (Fig. 4D). For the virus and PeptiCRAd groups ELISpot showed a strong immune response against viral stimulation. For the SIINFEKL peptide (Fig. 4E), the PeptiCRAd group also exhibited a robust T-cell response, significantly higher than the other groups.

These results confirm that the PeptiCRAd vaccine effectively induces a strong cellular immune response, crucial for long-term tumor control and prevention of recurrence. This comprehensive analysis demonstrates the exceptional potential of PeptiCRAd delivered via MNs in eliciting robust anti-tumor immune responses and achieving complete tumor rejection. The data underscores the superiority of this delivery method over traditional approaches, making it a promising candidate for cancer immunotherapy.

### 3.6 Enhanced cDC1 population and effector T cell memory triggered by transdermal vaccination

**Fig. 5:**
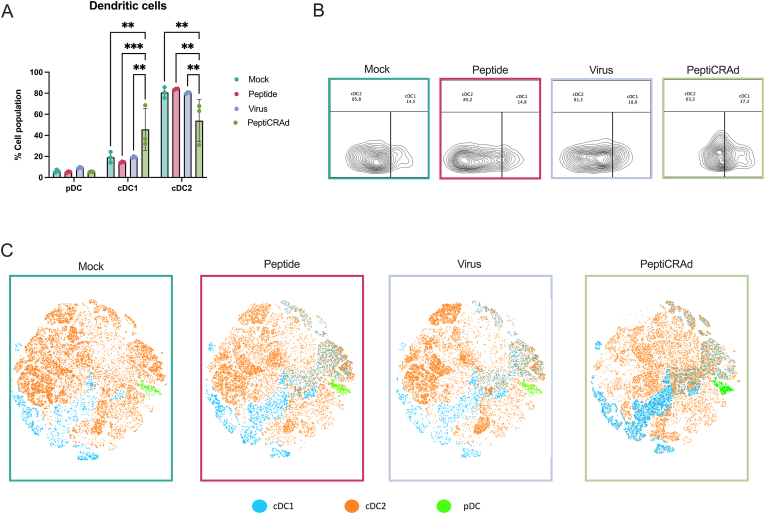
**Assessment of dendritic cell subsets post PeptiCRAd MN vaccination.** (A) Quantification of dendritic cell subsets, showing an increased percentage of cDC1 cells and a decrease in cDC2 cells in the MN containing PeptiCRAd group compared to other groups. (B) Contour plots from flow cytometry analysis depicting the distribution of cDC1 and cDC2 cells across different treatment groups. (C) t-SNE maps illustrating the higher number of cDC1 populations in the MN containing PeptiCRAd group. Statistical analysis was conducted using ANOVA, with p-values set at ∗p < 0.05, ∗∗p < 0.01, ∗∗∗p < 0.001, and ∗∗∗∗p < 0.0001.

After observing complete tumor rejection in the MN containing PeptiCRAd group, we proceeded to analyze the draining lymph nodes and spleens from all mice for further assessment of the elicited immune responses. We particularly focused on the dendritic cell (DC) subsets addressing the compartment potentially present in the viable skin. While plasmacytoid dendritic cells (pDCs) were found to be present at very low frequency, between 2 and 5%, in all the groups, notably, the MN containing PeptiCRAd group had a higher percentage of conventional cDC1 at 40 % compared to approximately 20 % in the other groups (Fig. 5A). This increase was associated with a decrease in cDC2 levels in the MN containing PeptiCRAd group (Fig. 5B). To further investigate these findings, we plotted a t-SNE map based on the DC populations and observed that the MN containing PeptiCRAd group had a higher number of cDC1 populations compared to the other groups (Fig. 5C). These results indicate a switch from cDC2 to cDC1, which is essential for MHC-I antigen presentation required for SIINFEKL peptides. This shift suggests a more vigorous immune response activation, which indicates the involvement of such subsets in the observed tumor rejection.

**Fig. 6:**
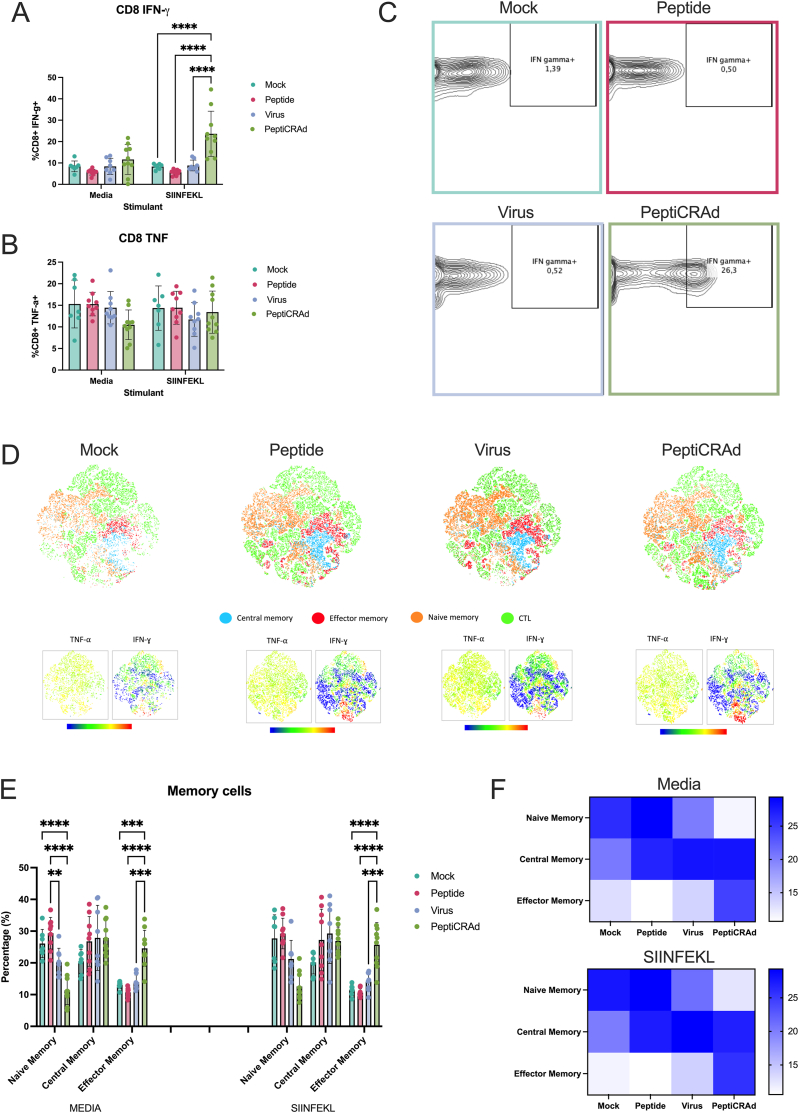
**Analysis of T Cell Responses in PeptiCRAd MN Vaccinated Mice.** (A) Quantification of IFN-g release by CD8^+^ T cells after stimulation with media or SIINFEKL. (B) Quantification of TNF-a release by CD8^+^ T cells. (C) Contour plots from flow cytometry analysis depicting IFN-g producing CD8^+^ T cells. (D) t-SNE plots illustrating the distribution of CD8^+^ T cell populations in different treatment groups. (E) Quantification of memory T cell phenotypes, showing an increase in effector memory T cells and a decrease in naïve memory T cells in the MN containing PeptiCRAd group. (F) Heat map representing the memory T cell phenotypes in different treatment groups. Statistical analysis was conducted using ANOVA, with p-values set at ∗p < 0.05, ∗∗p < 0.01, ∗∗∗p < 0.001, and ∗∗∗∗p < 0.0001.

After observing a stimulation in cDC1, we sought to investigate the T cell response elicited in each group. Splenocytes from all groups were collected and pulsed with either media or SIINFEKL. Using Golgi and ER inhibitors, we analyzed the release of IFN-g and TNF-α after a 6-h stimulation with either stimulant. With media treatment, no statistically significant differences were observed in IFN-g or TNF-a levels among CD8^+^ T cells across groups, with baseline levels of 10 % and 15 %, respectively. Interestingly, SIINFEKL treatment resulted in a significant increase in IFN-γ from 10 % to 20 % specifically in the MN containing PeptiCRAd group, while no increase was observed for TNF-α. This suggests a potent T cell immune response against SIINFEKL in the MN-treated group, potentially due to the skin-targeted delivery of the antigen via MNs, which ensures direct contact with cDC1 in the skin. cDC1 is particularly efficient in cross-presenting antigens to CD8^+^ T cells, a critical mechanism for robust IFN-γ release and tumor-specific immune activation. This enhanced T cell response may explain the complete tumor rejection observed in this group (Fig. 6A–C).

To further investigate the above, we analyzed T cell memory phenotypes upon stimulation with media or SIINFEKL. Mice given MN containing PeptiCRAd exhibited an overall increase in effector memory T cells when treated with both media and SIINFEKL. This indicates the formation of a baseline effector memory T cell population in mice treated with MN containing PeptiCRAd. The observed lower levels of naïve memory T cells in the MN-treated group also support this finding, as a higher proportion of T cells transitioned to effector memory status. tSNE plots of CD8^+^ T cells treated with SIINFEKL further revealed that the increased IFN-γ production originated from both non-memory cytotoxic T cells and effector memory T cells, highlighting the strong activation and differentiation of these cells in response to MN-based PeptiCRAd delivery (Fig. 6D–F). These data indicate that MN delivery with PeptiCRAd promotes a robust effector memory T cell response towards SIINFEKL, likely due to the specific activation of cDC1 populations. cDC1's role in MHC-I cross-presentation may facilitate a more targeted and sustained T cell response, enhancing the therapeutic efficacy of MN-mediated cancer immunotherapy.

## 4 Discussion

Recent studies have shown that MNs not only reduce pain but also increase patient compliance, which is crucial for the success of vaccination programs [40]. For instance, a phase 1/2 trial of a measles and rubella vaccine in the form of MN-patches showed good tolerability, safety and immunogenicity in children, pointing to the possibility of translating this delivery system for cancer vaccines [41]. The present work also underlines the potential of MNs in oncology for the administration of the PeptiCRAd vaccine. There was a significant rise in effector memory T-cells and CD8^+^ T-cells with IFN-g in the PeptiCRAd vaccinated group. This shows that the vaccine could stimulate a long-term anti-tumor immune response. Such responses are important in management of the tumor and prevention of tumor relapse [42]. Therefore, this vaccine-based MN-technology is well-suited for vaccines targeting cancers with a high relapse rate.

Several studies further support the potential of MN-technology to improve therapeutic outcomes and patient adherence in cancer immunotherapy [43]. Our findings confirm that MNs are effective and can efficiently administer cancer vaccines, providing a viable option to the conventional methods of immunization. This approach not only can increase patient satisfaction but also can increase the effectiveness of the vaccination programs and open new opportunities for the use of the vaccination in the clinic. Effector memory T-cells (TEM) are crucial for the immediate response to the re-exposure to antigens, which underlines the significance of both the acute and chronic immune responses in cancer treatment [44]. Recent studies have demonstrated that the presence of TEM cells within the tumor microenvironment is crucial for the efficacy of immune checkpoint blockade therapies. These cells exhibit diverse proinflammatory effector functions upon secondary antigen encounter, facilitating a robust and sustained immune attack on cancer cells [45]. The heterogeneity and functional plasticity of TEM cells have also been highlighted as key factors in determining the clinical outcome of cancer immunotherapies, further underscoring their importance in long-term immune surveillance and tumor control [46].

The dual functionality of the PeptiCRAd vaccine delivered via MNs – potent initial immune response and memory response – underscores its potential in future cancer vaccines. Critical to the success of our approach is the role of cDC1s. These cells are essential for cross-presenting antigens to CD8^+^ T cells, thereby initiating a robust cytotoxic T-cell response [47]. Our study provided further insights into the vaccine delivery and activation of dendritic cells and the expansion of TEM cells [43,[48], [49], [50]]. By enhancing the function of cDC1s, PeptiCRAd transdermal vaccination ensures a more effective and targeted immune attack on tumor cells. Recent research has highlighted the importance of cDC1s in priming CD8^+^ T-cell responses, with studies demonstrating their critical role in the cross-presentation of tumor antigens and subsequent T-cell activation [51,52]. Dissolving MNs degrade safely within the skin with no need for removal, reducing biohazard waste and this is crucial for patient compliance and environmental sustainability. We used sucrose to enhance the encapsulation of biological drugs, significantly improving stability and efficacy. The potential of using swelling MNs, or a combination of dissolving and swelling MNs, holds promise for controlled release over time.

## 5 Conclusion

The PeptiCRAd platform's modular nature allows rapid customization for various infectious diseases by altering surface peptides. While PeptiCRAd is designed for cancer immunotherapy, its adaptability is crucial for emerging infectious threats. Speed and flexibility are essential. PeptiCRAd-based vaccines could offer comprehensive protection against infectious diseases by eliciting strong T-cell responses, critical for long-lasting immunity. Our study highlights the potential versatility of PeptiCRAd beyond oncology. For example, a system called PeptiVAX was developed using the same platform to address SARS-CoV-2 [53]. By identifying CD8 T-cell epitopes from conserved regions of coronaviruses and creating peptides presented by common HLA-Is, PeptiVAX broadened T-cell responses beyond the SPIKE protein. This demonstrates our platform's capability to adapt rapidly to new infectious threats, emphasizing its broad applicability.

The success of mRNA vaccines during the COVID-19 pandemic demonstrated the importance of flexible and scalable vaccine platforms [54]. Similarly, the surface peptides of PeptiCRAd can be easily changed to achieve quick customization. This would make it very suitable for many infectious diseases, improving the global vaccine approach, especially for the new threats where speed and flexibility are of the essence. However, challenges remain. The issues that include scalability of MN production, the stability of MNs in the long run, and the legal issues that may affect the production of MNs must be discussed. The production of MNs with a standard quality and performance at a large scale is still challenging. The stability of MNs vaccines and their payload must be studied to confirm the effectiveness after long term storage. It is crucial to engage with regulatory authorities to facilitate the approval of these technologies and get them to the patients [[55], [56], [57]]. Advances in manufacturing technologies, such as 3D printing and automated production lines, could help reduce costs and make MNs more accessible [58]. Exploring partnerships with pharmaceutical companies and government agencies could provide necessary support for large-scale production and distribution.

In summary, MN-technology shows significant potential to improve cancer prevention and broader immunotherapy strategies. Our study confirms that MNs can effectively deliver the PeptiCRAd vaccine, inducing a robust anti-tumor immune response. This vaccine-based MN-technology is likely to enhance patient compliance due to its minimally invasive nature, making it more acceptable for long-term cancer prevention strategies. MNs deliver vaccines directly to the skin's immunologically active layers, maximizing immune activation while minimizing systemic exposure and side effects. Thus, our work emphasizes the combination of MN-technology with platforms like PeptiCRAd, which opens a new perspective for cancer immunotherapy with high accuracy and patient comfort. This work on MNs and PeptiCRAd opens possibilities for further advancements for cancer therapy.

## CRediT authorship contribution statement

**Carmine D'Amico:** Writing – review & editing, Writing – original draft, Visualization, Validation, Software, Methodology, Investigation, Funding acquisition, Formal analysis, Data curation. **Manlio Fusciello:** Writing – review & editing, Writing – original draft, Visualization, Validation, Software, Methodology, Investigation, Formal analysis, Data curation, Conceptualization. **Firas Hamdan:** Writing – review & editing, Visualization, Validation, Methodology, Investigation, Formal analysis, Data curation. **Federica D'Alessio:** Writing – review & editing, Investigation. **Paolo Bottega:** Writing – review & editing, Investigation. **Milda Saklauskaite:** Writing – review & editing, Investigation. **Salvatore Russo:** Writing – review & editing, Investigation. **Justin Cerioni:** Writing – review & editing, Investigation. **Khalil Elbadri:** Writing – review & editing, Investigation. **Marianna Kemell:** Writing – review & editing, Visualization, Investigation, Formal analysis. **Jouni Hirvonen:** Writing – review & editing, Supervision. **Vincenzo Cerullo:** Writing – review & editing, Supervision, Project administration, Funding acquisition, Conceptualization. **Hélder A. Santos:** Writing – review & editing, Supervision, Resources, Project administration, Funding acquisition, Conceptualization.

## Ethics approval and consent to participate

All animal experiments were reviewed and approved by the Experimental Animal Committee of the University of Helsinki and the Provincial Government of Southern Finland (license numbers ESAVI/11895/2019 and ESAVI/12722/2022). The maximal tumor size/burden allowed by our ethical permit is 18 mm in diameter. This limit was never exceeded in any of the experiments conducted in this study.

## Funding sources

The authors acknowledge the support from the Research Council Finland (grant No. 331151, HAS), 10.13039/501100005075UMCG Research Funds (HAS), and the 10.13039/501100003125Finnish Cultural Foundation (10.13039/100011639CD). Additional funding was provided by the 10.13039/501100000781European Research Council (ERC) under the 10.13039/501100007601Horizon 2020 framework (grant No. 681219, 10.13039/100026939VC), the 10.13039/501100004155Magnus Ehrnrooth Foundation (project No. 4706235, 10.13039/100026939VC), and the 10.13039/501100004012Jane and Aatos Erkko Foundation (project No. 4705796, 10.13039/100026939VC). The authors also acknowledge the Finnish Cancer Foundation (project No. 4706116, VC), the Helsinki Institute of Life Science (HiLIFE) (project No. 797011004, VC), the Digital Precision Cancer Medicine Flagship iCAN (VC), and the GeneCellNano Flagship (VC).

## Declaration of competing interest

The authors declare the following financial interests/personal relationships which may be considered as potential competing interests. Hélder A. Santos reports a relationship with Research Council Finland and UMCG that includes: funding grants. Carmine D'Amico reports a relationship with 10.13039/501100003125Finnish Cultural Foundation that includes: funding grants. Vincenzo Cerullo reports a relationship with 10.13039/501100000781European Research Council (ERC), 10.13039/501100004155Magnus Ehrnrooth Foundation, 10.13039/501100004012Jane and Aatos Erkko Foundation, Finnish Cancer Foundation that includes: funding grants. If there are other authors, they declare that they have no known competing financial interests or personal relationships that could have appeared to influence the work reported in this paper.
