# Supplementary material for: Transdermal delivery of PeptiCRAd cancer vaccine using microneedle patches
Source: Bioact Mater. 2024 Nov 19;45:115–27. doi: 10.1016/j.bioactmat.2024.11.006 (PMC11617629; doi:10.1016/j.bioactmat.2024.11.006)
Supplement: Multimedia component 1 [file mmc1.docx]

**Supporting Materials**


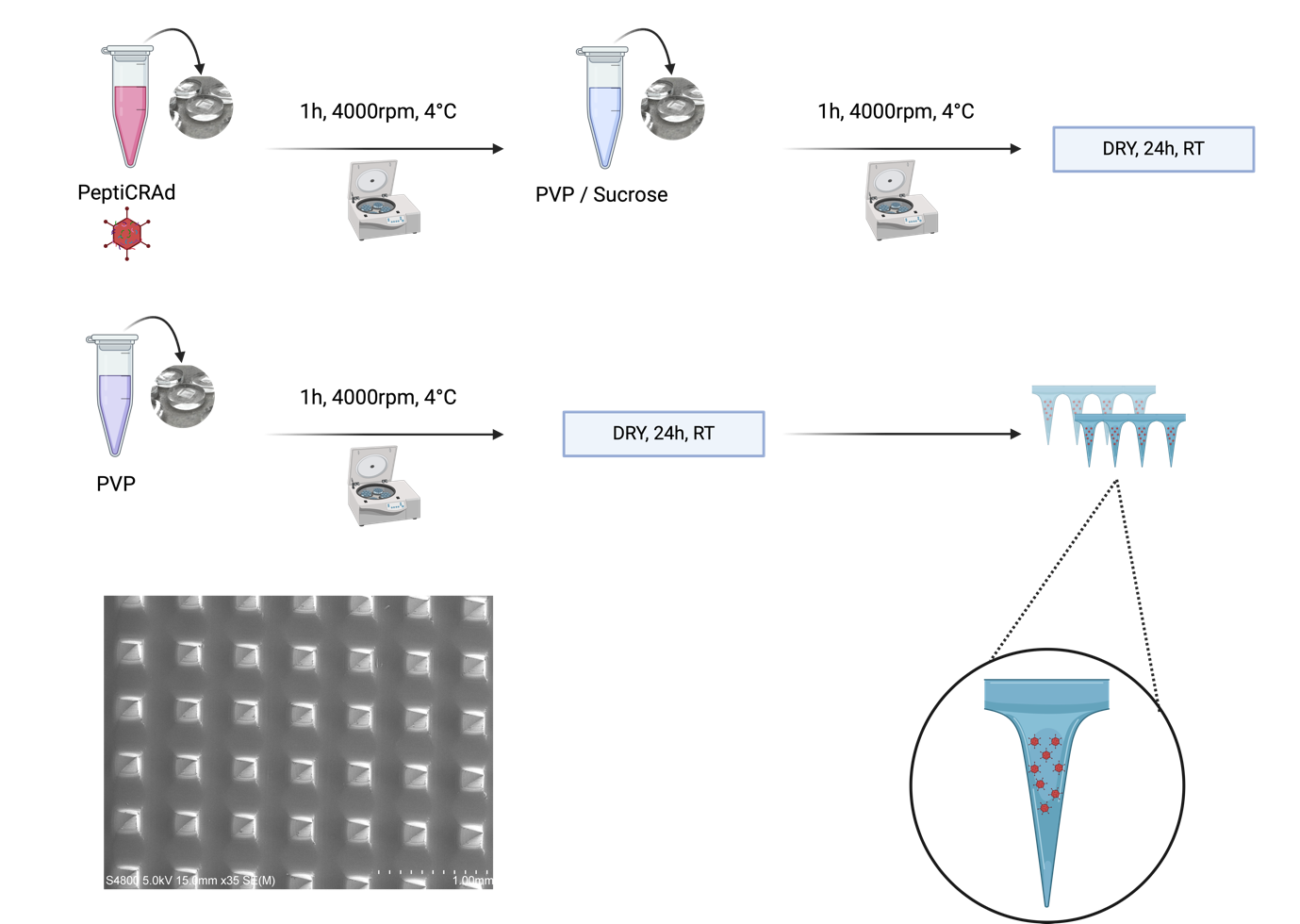


Figure S1: Schematic representation of the manufacturing process for MNs using controlled temperature micro-molding.

Figure S2: Comparison of piercing abilities through multiple layers of Parafilm (approximately 100 µm thick each) to ensure that sucrose and PeptiCRAd addition does not compromise mechanical properties or penetration capability.

**Drug Loading Efficiency and Infectivity Quantification of PeptiCRAd MNs**

To assess virus loading efficiency in PeptiCRAd-loaded microneedles (MNs), the viral particle concentration was initially measured at 260 nm to determine particle counts. Infectious units (IUs) were quantified by immunocytochemistry (ICC) using A549 cells as the infection model. In this procedure, MNs were dissolved in PBS, and the resulting solution was applied to A549 cells. Following infection, cells were fixed and permeabilized, then stained specifically for the viral hexon protein to identify infected cells. Positive cells were visualized and counted under a fluorescence microscope. The positive control, representing the viral particles used before MN encapsulation (without any losses), was used for comparison. Results were expressed as a percentage of infectivity relative to this positive control, allowing for accurate quantification of any loss in viral infectivity during the MN preparation and loading process.

Figure S3: ICC quantifying the loss during the loading of PeptiCRAd within the microneedles. Cells were stained to visualize the viral presence, with fluorescence indicating effective viral loading and retention in the MN formulation.
